# Life History Parameters of the Sunn Pest, *Eurygaster integriceps*, Held at Four Constant Temperatures

**DOI:** 10.1673/031.010.10601

**Published:** 2010-07-13

**Authors:** Shahzad Iranipour, Aziz Kharrazi Pakdel, Gholamreza Radjabi

**Affiliations:** ^1^ Department of Plant Protection, Faculty of Agriculture, University of Tabriz, Tabriz, Iran; ^2^ Department of Plant Protection, Faculty of Agriculture, University of Tehran, Karaj, Iran; ^3^ Plant Pests and Diseases Research Institute, P.O. Box 1454-19395 Tehran, Iran

**Keywords:** degree-day, intrinsic rate of increase, net replacement rate

## Abstract

The Sunn-pest, *Eurygaster integriceps* Puton (Heteroptera: Scutelleridae), is the most important insect pest of wheat and barley in Iran. A demographic study was carried out in order to determine the effect of temperature on life history parameters of the pest. Life tables were constructed at four constant temperatures: 22, 25, 27, and 30 ± 1° C using Mahdavi wheat kernels as food. Finite and intrinsic rates of population increase, gross and net reproductive rates, intrinsic rates of birth and death, generation time, doubling time, and lifetime female fecundity all varied significantly among temperatures. The intrinsic rate of natural increase, r_m_, increased linearly with temperature and was estimated to be 0.0126, 0.0381, 0.0541, and 0.0789 females/female/day, respectively, at the above-mentioned temperatures. Generation time ranged from 121 days at 22° C to 40 days at 30° C. Net replacement rate was significantly lower at 22° C than at other temperatures (4.6 vs. 22.2 to 25.8 females/female/generation). Lifetime female fecundity ranged from 123.1 at 22° C to 209.4 at 30° C. The thermal threshold for post-diapause pre-reproductive development was estimated to be 20° C, and 66.8 degree-days were required for its completion.

## Introduction

The Sunn pest, *Eurygaster integriceps* Puton (Heteroptera: Scutelleridae), is the most important insect pest of cereals (wheat and barley) in Iran. It has a single generation a year and an obligatory diapause in the adult stage (Alexandrov 1947; Radjabi 2000). Despite a long history of the pest in Iran, there are few studies, if any, on the effects of environmental factors on its demography. Some work has addressed the effect of different wheat cultivars on biological parameters like fecundity (Zomorrodi 1961; Abdollahy 1989b; Karimi 1992; Rezabeygi et al. 2000; Fathipour and Abdollahy 1998) and reproductive diapause (Martin et al. 1969; Abdollahy 1989a; Radjabi and Termeh 1992; Radjabi 2000). The average number of eggs laid under field conditions has been reported to be 70–80/female (Alexandrov 1947; Davatchi 1954). Zomorrodi (1961) found that feeding on wheat kernels caused a 3-fold increase in oviposition compared to feeding on seedlings. On the other hand, fecundity declined with increasing density in laboratory cultures.

Martin et al. (1969) examined oviposition in *E. integriceps* as a function of diapause status. They observed that samples obtained from resting sites between October and April laid 0.1 to 11.5 clutches of eggs/female. They also observed a negative effect on oviposition in crowded cultures. Abdollahy (1989a) found that adults collected during the first week of November, December, and January laid 53.5, 75 and 100% of their eggs, respectively. Similarly, Iranipour (2008) observed oviposition by 62 and 86% of females collected during December and February, respectively, with mean fecundities of 110.5 and 262.5, respectively. Radjabi (2000) emphasized that fecundity decreases after January due to a decline in fat reserves. Safavi (1973), Salavatiyan (1991), and Radjabi (1994, 2000, 2007) suggested that *E. integriceps* can be divided into two categories by weight: the first group includes aggressive or virulent bugs, which have a weight over 125 mg, whereas the second group comprises avirulent bugs that are below 110 mg and lay only 50 to 70% as many eggs as the first group. These groups are also recognized as migrant and resident bugs, respectively (Radjabi 2000, 2007). Whereas consistent differences in weight and fecundity are often evident between populations, Iranipour (2008) found no within-group correlation between body size and fecundity, suggesting no direct relationship between the two.

Maafi and Parker (2001) and Neyshabouri (2006) measured demography of the pest in separate studies under constant physical conditions. However, despite the obvious effect of temperature on factors affecting population growth rate, no study has yet quantified its effects on mortality, natality, and developmental rate of the pest. In this study, four constant temperatures were used to examine the effects on fecundity, developmental time, and survival.

## Materials and Methods

### Source of insects

Adult *E. integriceps* were collected from their resting sites in Varamin County, Tehran Province during diapause termination in December and used directly in experiments. The obligatory diapause complicates rearing and makes it difficult to obtain a homogenous cohort.

### Experimental design

Adults were divided into four equal groups, each consisting of 15 pairs of males and females, each held in a plastic cylinder (12 cm diameter × 17 cm height). All insects were held in an insectary under physical conditions simulating natural spring conditions. Hence, four constant temperatures were tested: 22, 25, 27, and 30 ± 1° C, all with a photoperiod of 14:10 L:D. Bugs were fed on wheat kernels cultivar Mahdavi, a commonly cultivated variety in Varamin. The tops of dishes were covered with a cloth net, and the bottoms were pressed into soil in a pot with a similar internal diameter. The soil provided a textured substrate that was conducive to the bugs righting themselves when they fell on their backs. Food was replaced two to three times a week and water was provided continuously in a vial. Strips of paper (4 × 30 cm) were folded and placed in each cylinder as an oviposition substrate. All events were recorded daily throughout the experiment until the last individual died.

**Life tables.** The obligatory adult diapause renders it impractical to rear a cohort from egg to egg. Therefore, data on the development of immature stages were obtained in a separate experiment using egg cohorts collected at the peak of oviposition under the same conditions as described above (Iranipour et al. 2003). These data provided constant values for developmental time (egg to adult molt) and immature survival rate at each temperature. The following life table parameters were measured using the procedures of Carey (1993). Gross reproductive rate (GRR), net reproductive rate (Ro), intrinsic rate of natural increase (r_m_), finite rate of increase (λ), intrinsic birth rate (b), intrinsic death rate (d), generation time (GT), and doubling time (DT) were estimated for each temperature treatment. Jackknife pseudo-values were used to estimate variances and confidence intervals (Meyer et al. 1986). Additional variables such as lifetime fecundity/female, pre-oviposition, oviposition, and post-oviposition periods and longevity were analyzed by traditional methods using the GLM procedure of SAS (1999). Due to unequal mortality and infertility among treatments, statistical analysis assumed an imbalanced CRD. Means were separated using Duncan's multiple range test at both 95 and 99% confidence levels, except for lifetime fecundity for which Tukey's procedure was used. The linear regression model PROC REG (SAS 1999) was used to describe the relationship between developmental rate and temperature.

## Results

### Demographics

**Figure 1.**
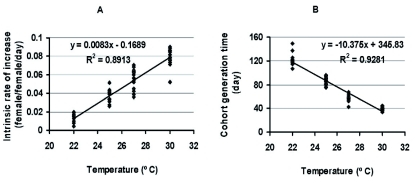
Relationship between temperature and *Eurygaster integriceps* intrinsic rate of natural increase (A) and generation time (B). High quality figures are available online.

Significant differences were observed in all life history parameters across the range of temperatures tested (Table 1). All life history parameters increased in magnitude with increasing temperature, except for generation time and doubling time which demonstrated the opposite trend. There was a significant differences among temperatures in all dependent variables (p < 0.01 in all cases). The relationship between temperature and r_m_ (*F* = 475.72; df = 1, 58; p < 0.01; r^2^ = 0.891) was positive, whereas that between temperature and T (*F* = 749.05; df = 1, 58; p < 0.01; r^2^ = 0.928) was negative (Figure 1).

### Lifetime fecundity

**Figure 2.**
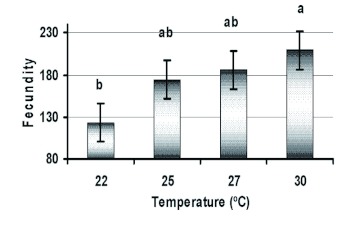
Mean fecundities of fertile females of *Eurygaster integriceps* held at four different temperatures. Columns bearing the same letter were not significantly different (Tukey's HSD, α = 0.05). High quality figures are available online.

Although ANOVA revealed no significant difference among temperatures whether all females (*F* = 1.795; df = 3, 56; p = 0.16) or only fertile females (*F* = 2.553; df = 3, 51; p = 0.07) were considered, Tukey's procedure revealed a significant difference between 22 and 30° C at the p <0.05 (Figure 2). Average fecundities increased to 209.4, 185.8, 174.3, and 123.1, respectively, when infertile females were eliminated.

### Longevity

**Figure 3.**
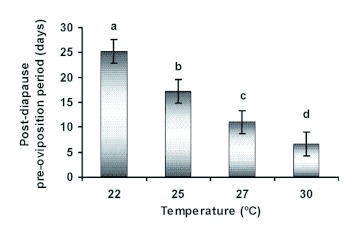
Mean post-diapause pre-oviposition periods of *Eurygaster integriceps* females held at four different temperatures. Columns bearing different letters were significantly different (Duncan's MRT, α = 0.01). High quality figures are available online.

The post-diapause adult life of females can be divided into three components, the preoviposition, oviposition, and post-oviposition periods. Effects of temperature on longevity and the duration of these periods were analyzed separately. Significant differences were observed in longevity (*F* = 18.97; df = 3, 56; p < 0.01). Longevity was greater when the bugs were reared at 22 and 25° C in comparison with 27 or 30° C. Mean longevities were 70.8 ± 5.1, 64.9 ± 5.0, 39.2 ± 4.5, and 30.3 ± 3.1 days at 22, 25, 27, and 30° C, respectively. Temperature significantly affected the post-diapause pre-oviposition (*F* = 63.43; df = 3, 51; p < 0.01) oviposition (*F* = 4.81; df = 3, 51; p = 0.01), and postoviposition periods (*F* = 3.60; df = 3, 51; p = 0.02). The post-diapause pre-oviposition period decreased with increasing temperature (Figure 3). The lower thermal threshold for reproductive development was estimated to be 20.0° C, and 66.8 degree-days were required for its completion.

**Table 1.**
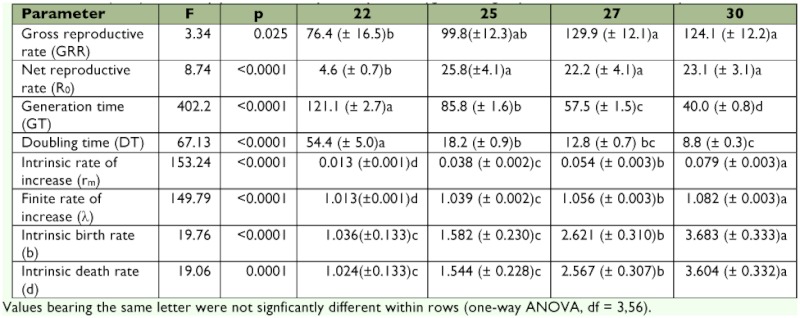
Mean (± SE) life history parameters for post-diapause *Eurygaster integriceps* adults held at four temperatures.

The oviposition period was significantly longer at 22 and 25° C than at 30° C, lasting 37.4 ± 3.9, 39.2 ± 5.4, 28.0 ± 4.5, and 19.6 ± 2.3 days at 22, 25, 27, and 30° C, respectively. The post-oviposition period was also longer at 22° C than at either 27 or 30° C, lasting 11.2 ± 2.1, 8.2 ± 1.7, 3.9 ± 0.8, and 5.7 ± 1.7 days at the four temperatures, respectively. Fecundity was positively correlated with longevity and oviposition period and negatively correlated with pre-oviposition period, whereas longevity was positively correlated with all other parameters (Table 2).

## Discussion

The relationship between weather conditions and *E. integriceps* outbreaks were pointed out by Kartavtsev (1974), Vinogradova and Kosenkov (1976), Doroninia and Makarova (1976), Makarova and Doroninia (1981), and Volodichev (1991, 1996). Studying *Eurygaster amerinda* and *Eurygaster minidoka* in Pachkopas and Berkley, Vojdani (1961) showed that population changes from one generation to the next were independent of pest density in the previous generation and that weather conditions were responsible for most of the observed variation. Rainfall patterns during the two years were inferred to affect phenological synchrony of the pest with its host plant. Subsequently, other workers reemphasized the indirect effect of drought on pest abundance via its indirect effect on wheat growth and nutrition (Safavi 1971, 1972; Salavatiyan 1991). Rainy, cold springs also may have a similar effect due to differences in thermal thresholds for *E. integriceps* versus wheat growth and development (Fedotov 1954; Paikin 1969; Salavatiyan 1991). Afsarian et al. (2006) found that drought may also cause direct mortality in the pest population. When fields were irrigated for longer periods, densities of nymphs and adults in the next generation were lower even when initial adult densities were the same. The thermal threshold for immature development of *E. integriceps* was determined by Iranipour et al. (2003) who reported that developmental time decreased from 77.7 to 24.5 days as temperature increased from 22 to 30° C. This is the primary reason why the intrinsic rate of natural increase r_m_ was observed to increase with temperature in the present study. As pointed out by Lewontin (1965) and Dent and Walton (1997), r_m_ is affected more by age of first reproduction than by fecundity. Delayed development causes a delay in onset of reproduction and a parallel increase in generation time.

Maafi and Parker (2001) studied demography of this insect at 25 ± 0.5° C, and obtained results that differed from those presented here, including lower GRR, higher R_0_ and r_m_, shorter GT and DT, and lower overall mortality than the present results at the same temperature. The more than 4-fold difference in GT between the two studies reflects parallel differences in DT and r_m_. The shorter developmental time in their study (41.56 vs. 51.47) and lower mortality (40% vs. 66%), as well as unknown differences in methodology may explain some of these differences. Although the sources of two populations were the same, the generations were different and that of their study appeared more vigorous, developing faster with lower mortality. This may arise from differences in the physiological state of the pest population in different years or from maternal effects carried over from the previous generation. However, in the Maafi and Parker (2001) study, data on immature stages were not included in the calculation of GT. If development time is taken into account in calculating generation time, then there is a 3fold increase in GT in the Maafi and Parker (2001) study.

**Table 2.**
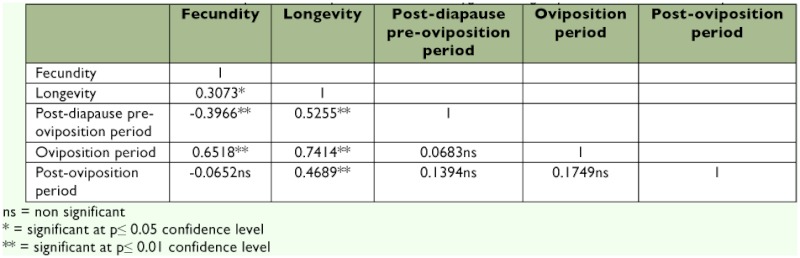
Correlations between components of reproductive life in *Eurygaster integriceps* adults held at four temperatures.

Neyshabouri (2006) compared the demographics of normal bugs with melanic morphs and found no differences between them except for developmental time, which was about a day longer for melanics. Those results were similar to the current data, which was obtained at 27° C in both cases.

Radjabi (2000, 2007) observed that cold and rainy conditions in spring could delay development of *E. integriceps* in relation to wheat. Moreover, oviposition begins later and is extended over a longer period. This has an indirect effect on the next generation by shortening its feeding window. Salavatiyan (1991) also stated that, when such conditions occur in successive years, they can lead to a considerable decline in the population. Vojdani (1961) observed both temporal and spatial differences in developmental time in two Nearctic species of *Eurygaster*. However, the population was affected negatively by increasing temperature in his study because direct mortality due to high temperatures was considerable, and the adults left the fields after emergence. It is clear from the current results that the best reproduction of the insect occurred at temperatures ≥ 25° C and that at 30° C, the upper temperature threshold for reproduction was not yet reached. Shorter developmental times at higher temperatures further enhanced the intrinsic rate of natural increase within the range studied. From this, it may be inferred that *E. integriceps* will be more harmful to cereal crops during a hot spring than a cold one as a result of both accelerated development and faster reproduction.
